# Race, Ethnicity, and Nasopharyngeal Cancer Subtypes in the US

**DOI:** 10.1001/jamanetworkopen.2025.51219

**Published:** 2026-01-12

**Authors:** Asher Shin, Stephanie Wang, Jennifer Ma, Shivaek Venkateswaran, Rohit V. Mantena, James O. Suggitt, Yingzhi Wu, Erin Jay G. Feliciano, Luisa E. Jacomina, Irini Yacoub, Edward Christopher Dee

**Affiliations:** 1 Department of Radiation Oncology, Memorial Sloan Kettering Cancer Center, New York, New York; 2 Harvard College, Cambridge, Massachusetts; 3 College of Arts and Sciences, University of Pennsylvania, Philadelphia; 4 School of Medicine, University of Pittsburgh, Pittsburgh, Pennsylvania; 5 NYU Grossman School of Medicine, New York, New York; 6 Department of Medicine, NYC Health + Hospitals/Elmhurst, Icahn School of Medicine at Mount Sinai, Queens, New York; 7 Department of Radiation Oncology, University of Santo Tomas Hospital, Manila, Philippines; 8 New York Proton Center, New York

## Abstract

This cross-sectional study assesses associations of race and ethnicity with histologic subtype in US patients with nasopharyngeal cancer.

## Introduction

The burden of nasopharyngeal carcinoma (NPC) demonstrates geographic and racial and ethnic heterogeneity. In endemic areas, including Southern China, Southeast Asia, and North Africa, nonkeratinizing subtypes, associated with early-life Epstein-Barr virus (EBV) infection, account for most cases and generally have better outcomes.^1,2,3^ In nonendemic settings, such as the US, the less EBV-dependent keratinizing subtype, which carries a poorer prognosis, represents a larger burden of NPC.^1,2^ Whether this global pattern is replicated across the many racial and ethnic communities of the contemporary US population is unclear.^3^ Because histologic subtype may influence screening strategies, treatment selection, and survival, delineating distribution is essential to informing equitable, evidence-based care.

## Methods

The National Cancer Database (approximately 70% of US cancer diagnoses) was queried for NPC cases diagnosed from 2004 to 2021. Histologic subtypes, coded with *ICD-O-3*, were grouped into carcinoma not otherwise specified (NOS; code 8010), undifferentiated carcinoma (code 8020), squamous cell NOS (code 8070), keratinizing squamous cell (code 8071), and nonkeratinizing squamous cell (code 8072). Self-identified race and ethnicity were disaggregated. Five-year overall survival was quantified using the Kaplan-Meier method and multivariable Cox models adjusting for age, sex, stage, Charlson-Deyo score, and diagnosis year. Logistic regression evaluated race and ethnicity and keratinizing vs nonkeratinizing histologic subtype. Stata, version 16.1 (StataCorp) was used; 2-sided *P* < .05 signified statistical significance. Given publicly available National Cancer Database data, this research did not constitute human participants research and informed consent was not required. The STROBE reporting guideline was followed.

## Results

Among 22 807 patients with NPC (median [IQR] age, 57 [48-66] years; 15 942 [69.9%] male and 6865 [30.1%] female), histologic subtypes included squamous cell carcinoma NOS, 8442 (37.0%); nonkeratinizing, 4516 (19.8%); carcinoma NOS, 3721 (16.3%); keratinizing, 1452 (6.4%); and undifferentiated carcinoma, 1270 (5.6%). Compared with carcinoma NOS, overall survival was increased for undifferentiated carcinoma (hazard ratio [HR], 0.83; 95% CI, 0.74-0.92) and nonkeratinizing tumors (HR, 0.87; 95% CI, 0.81-0.94) and decreased for squamous NOS (HR, 1.33; 95% CI, 1.25-1.42) and keratinizing tumors (HR, 1.59; 95% CI, 1.45-1.74); results were similar in the 2012 to 2021 subset. The Table gives 5-year survival estimates for keratinizing, nonkeratinizing, and undifferentiated tumors.

**Table.** Five-Year Overall Survival for Keratinizing and Nonkeratinizing Nasopharynx Cancer in the National Cancer Database, 2004 to 2021

Cancer type	Estimate of patients alive at 5 years (95% CI)				
	Total	Stage I disease	Stage II disease	Stage III disease	Stage IV disease
Keratinizing (type 1)	43.0 (40.2-45.9)	53.3 (42.2-63.3)	66.6 (58.6-73.4)	42.5 (36.8-48.0)	33.9 (29.8-38.1)
Nonkeratinizing (type 2)	65.9 (64.3-67.5)	73.8 (67.6-79.0)	82.2 (78.8-85.1)	71.1 (68.2-73.7)	54.3 (51.6-57.0)
Undifferentiated (type 3)	70.0 (67.3-72.5)	79.0 (69.0-86.1)	86.8 (81.5-90.7)	78.3 (73.7-82.3)	54.0 (50.1-59.3)

Nonkeratinizing disease was most prevalent in Chinese (395 of 1411 [28.0%]), Hawaiian (19 of 66 [28.8%]), Indian or Pakistani (64 of 226 [28.3%]), Korean (26 of 82 [31.7%]), Thai (13 of 37 [35.1%]), and other Asian (266 of 849 [31.3%]) patients and less common in American Indian or Alaska Native (24 of 103 [23.3%]), Black (696 of 2944 [23.6%]), Japanese (7 of 49 [14.3%]), and White (2501 of 11 831 [21.1%]) groups. Keratinizing NPC was most common among American Indian or Alaska Native (8 of 103 [7.8%]), Black (206 of 2944 [7.0%]), Japanese (4 of 49 [8.2%]), and White (1110 of 11 831 [9.4%]) patients and was rare in the Kampuchean (0), Korean (1 of 82 [1.2%]), Laotian (1 of 82 [1.2%]), and Thai (0) cohorts (Figure).

**Figure.**
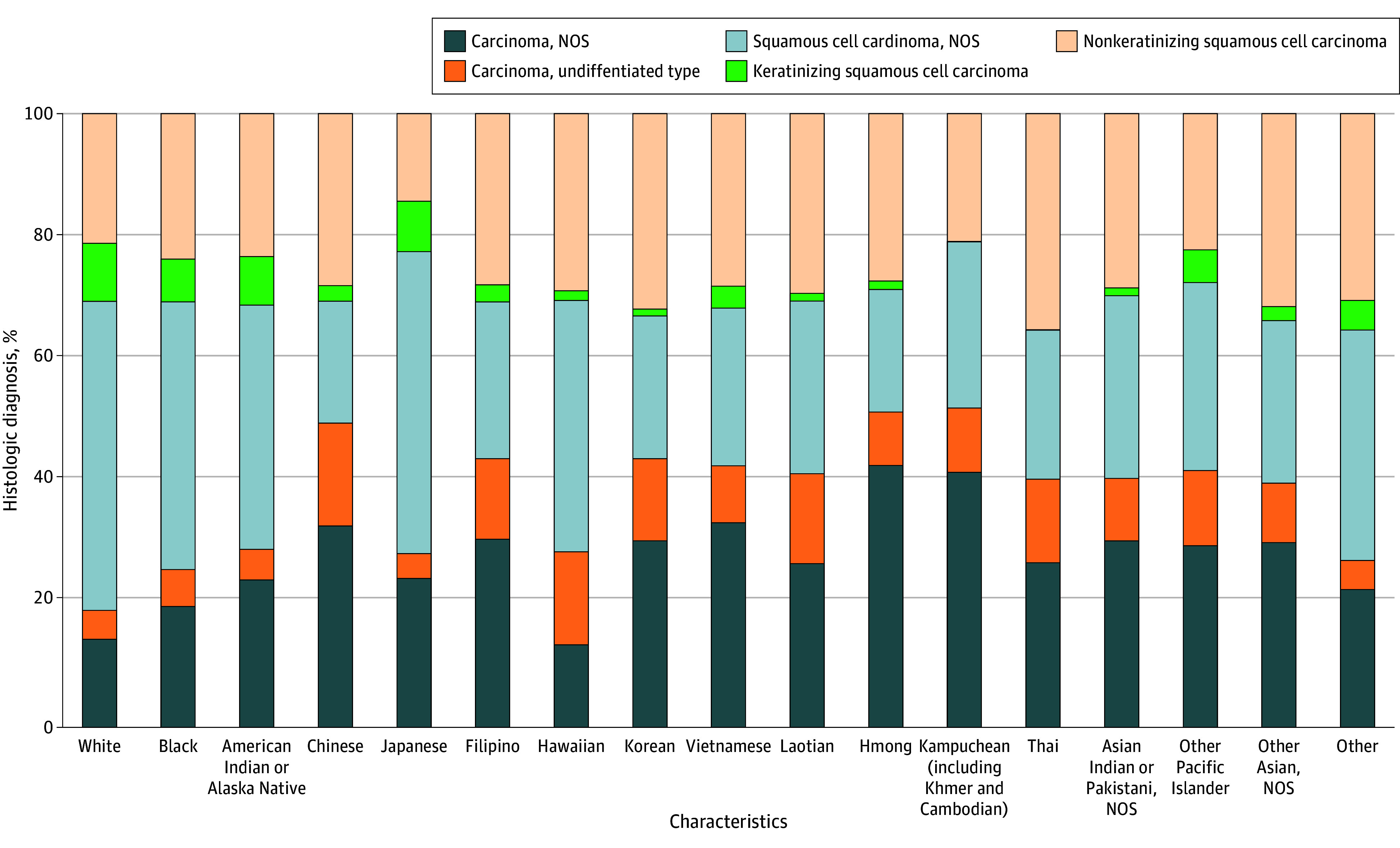
Distribution of the Most Common Histologic Subtypes of Nasopharynx Cancer Among Disaggregated Race and Ethnicity Groups in the US Among Patients Diagnosed From 2004 to 2021 NOS indicates not otherwise specified.

## Discussion

Our findings suggest that nonkeratinizing disease still dominates in many East Asian, South Asian, Southeast Asian, and Pacific Islander groups, possibly reflecting associations of risk factors such as EBV exposure, dietary factors, and genetic susceptibility. However, keratinizing NPC in Black, Japanese, American Indian or Alaska Native, and White populations may be associated with tobacco carcinogenesis, alcohol, and chronic mucosal inflammation.^1,2,3^ Clinically, keratinizing tumors relapse locally and generally confer worse survival, whereas nonkeratinizing tumors have a higher risk of distant metastasis and demonstrate a better response to radiotherapy and chemotherapy.^1,2,3^

Most NPC trials, largely conducted in endemic regions, overwhelmingly enroll nonkeratinizing cases.^4^ Our findings underscore the histologic diversity of NPC, calling into question the generalizability of these treatment paradigms to all racial and ethnic groups. Alaska Native, American Indian, and Black patients are more likely to present with keratinizing NPC and at more advanced disease stages,^5^ constituting populations often underrepresented in endemic-focused trials. Given higher mortality for keratinizing disease, future trials should oversample these groups and test intensified local and systemic therapies.^5,6^ Subtype-specific trials would prove insightful because trial paradigms from endemic countries may fail diverse US populations; the experience with human papillomavirus–stratified oropharyngeal cancer shows the feasibility of a subtype-specific paradigm.

Study limitations included retrospective design, missing smoking status, and lack of oncologic outcomes beyond overall survival. This study suggests keratinizing histologic subtype as an adverse prognostic factor and underscores pronounced racial-ethnic heterogeneity. Integrating histologic subtype into NPC research and treatment is essential for equitable care.

## Notes

Supplement.
Data Sharing Statement
