# Supplementary material for: Race, Ethnicity, and Nasopharyngeal Cancer Subtypes in the US
Source: JAMA Netw Open. 2026 Jan 12;9(1):e2551219. doi: 10.1001/jamanetworkopen.2025.51219 (PMC12797091; doi:10.1001/jamanetworkopen.2025.51219)
Supplement: Supplement. — Data Sharing Statement [file jamanetwopen-e2551219-s001.pdf]

Data Sharing Statement

Shin. Race, Ethnicity, and Nasopharyngeal Cancer Subtypes in the US. *JAMA Netw Open*. Published January 12, 2026. doi:10.1001/jamanetworkopen.2025.51219

Data
Data available: Yes
Data types: Deidentified participant data
How to access data: Data are available from the American College of Surgeons upon approved application.
When available: Data are accessible upon application to the American College of Surgeons.

Supporting Documents
Document types: Other (please specify)
Additional Information: Code
How to access documents: Reasonable request from corresponding author
When available: With publication

Additional Information
Who can access the data: Data are accessible upon application to the American College of Surgeons.
Types of analyses: Stata, NCDB
Mechanisms of data availability: Data are available from the American College of Surgeons upon approved application.
Any additional restrictions: n/a
